# Observational retrospective clinical study on clinical features of macrolide-resistant *Mycoplasma pneumoniae* pneumonia in Chinese pediatric cases

**DOI:** 10.1038/s41598-024-55311-2

**Published:** 2024-03-07

**Authors:** Peng Li, Wei Wang, Xianhui Zhang, Jie Pan, Lina Gong

**Affiliations:** 1 Department of Laboratory Medicine, Children’s Hospital of Shanxi Province, Taiyuan, China; 2 Department of Laboratory Medicine, Shanxi Provincial People’s Hospital, Taiyuan, China; 3 Department of Pathology, Stanford University School of Medicine, Palo Alto, CA 94305 USA; 4 Department of Medical Risk Management, The Third Medical Center of Chinese PLA General Hospital, Beijing, 100039 China

**Keywords:** *Mycoplasma pneumoniae*, Gene mutation, Antibiotic resistance, C-reactive protein, Interleukin-18, Community-acquired respiratory distress syndrome CARDS toxin, Microbiology, Clinical microbiology

## Abstract

This study aimed to investigate differences in clinical characteristics and laboratory findings between children infected with Macrolide-Sensitive *Mycoplasma pneumoniae* (MSMP) and Macrolide-Resistant *Mycoplasma pneumoniae* (MRMP). Additionally, the research sought to identify laboratory markers for rapidly distinguishing refractory *Mycoplasma pneumoniae* pneumonia (RMPP) from ordinary *Mycoplasma pneumoniae* pneumonia (OMPP). In total, 265 *Mycoplasma pneumoniae* (MP) patients were included, with MRMP identified by specific point mutations in domain V of the 23S rRNA gene. A retrospective analysis compared the clinical courses and laboratory data, revealing that MRMP patients experienced prolonged febrile days (*P* = 0.004), elevated CRP levels (*P* < 0.001), and higher MP DNA loads than MSMP patients (*P* = 0.037). Based on clinical symptoms, MRMP was divided into RMPP (n = 56) and OMPP (n = 70), with RMPP demonstrating significantly increased IL-18, community-acquired respiratory distress syndrome (CARDS) toxins in nasopharyngeal aspirate, and serum CRP levels (*P* < 0.001; *P* = 0.006; *P* < 0.001). In conclusion, timely recognition of RMPP is crucial for enhancing prognosis. The identification of MRMP, coupled with proinflammatory cytokines such as IL-18, CARDS toxins, and CRP, emerges as promising markers with the potential to contribute significantly to diagnostic accuracy and prognosis assessment.

## Introduction

*Mycoplasma pneumoniae* (MP) is a common pathogen of community-acquired pneumonia (CAP) in children, constituting 10–40% of CAP cases in school-aged children^1,2^. *Mycoplasma pneumoniae* pneumonia (MPP), usually responsive to macrolides, is considered self-limiting^3^. However, some children may develop refractory *Mycoplasma pneumoniae* pneumonia (RMPP), leading to increased attention in recent years^4–6^. The clinical symptoms and chest imaging are aggravated and persistent after being treated with macrolide antibiotics for 7 days or longer, which can be considered RMPP. RMPP can also cause a range of extrapulmonary manifestations, such as pericarditis, encephalitis, arthritis, hemolytic anemia, and even multiple organ dysfunction^3,7^. While the exact mechanism of RMPP remains largely unclear, but an excessive host immune response and infection by Macrolide-Resistant *Mycoplasma pneumoniae* (MRMP) are believed to contribute^2^.

The resistance of MP to macrolides was first described in Japan in 2001^8^. In recent years, there has been a steady rise in the reported occurrence of MRMP strains globally, although significant differences have been observed among countries. During epidemic years, the resistance rate in some countries has been found to exceed 90%^9,10^. As anticipated, this resistance diminishes the effectiveness of macrolides, resulting in persistent clinical symptoms, prolonged hospital stays, intensified antibiotic treatment, and worsened chest X-ray findings, collectively contributing to the development of RMPP^11–13^.

The challenging culture requirements and slow growth of MP make routine culture and phenotypic drug sensitivity testing insufficient for clinical use, primarily serving epidemiological purposes. Molecular techniques, particularly PCR, have significantly improved the sensitivity and specificity of MP infection diagnosis^14^. Macrolide resistance in MP is conferred by single base mutations in the V region of 23S rRNA, which encodes the binding site for macrolide. The most prevalent mutations involve the substitution of A with C/G/T at either location A2063 or location A2064^15,16^. In China, the A2063G mutation has emerged as the primary cause of macrolide resistance in MP, with a detection rate exceeding 95%^17,18^. Thus, detecting the A2063G and A2064G mutation in the V region of 23S rRNA could provide insights into the overall trend of Macrolide resistance to MP infection in children. Delay in receiving timely and appropriate treatment is associated with developing more severe or prolonged illnesses^2,19^. Therefore, enhanced clinical awareness and timely identification of MRMP can initiate effective treatment at an early stage, potentially improving clinical outcomes^20^. However, data on the clinical characteristics and laboratory findings of MRMP infections in children in China are still limited.

The aim of this study was to estimate the proportion of MRMP in a tertiary hospital in north China, and to investigate differences in clinical characteristics and laboratory findings between patients infected with MSMP and those with MRMP. MRMP was identified through the analysis of mutations within domain V of the 23S rRNA. Moreover, we aimed to identify potential laboratory markers that could be used in daily clinical practice to distinguish RMPP from ordinary *Mycoplasma pneumoniae* pneumonia (OMPP) quickly.

## Methods

### Study subjects

**Figure 1:**
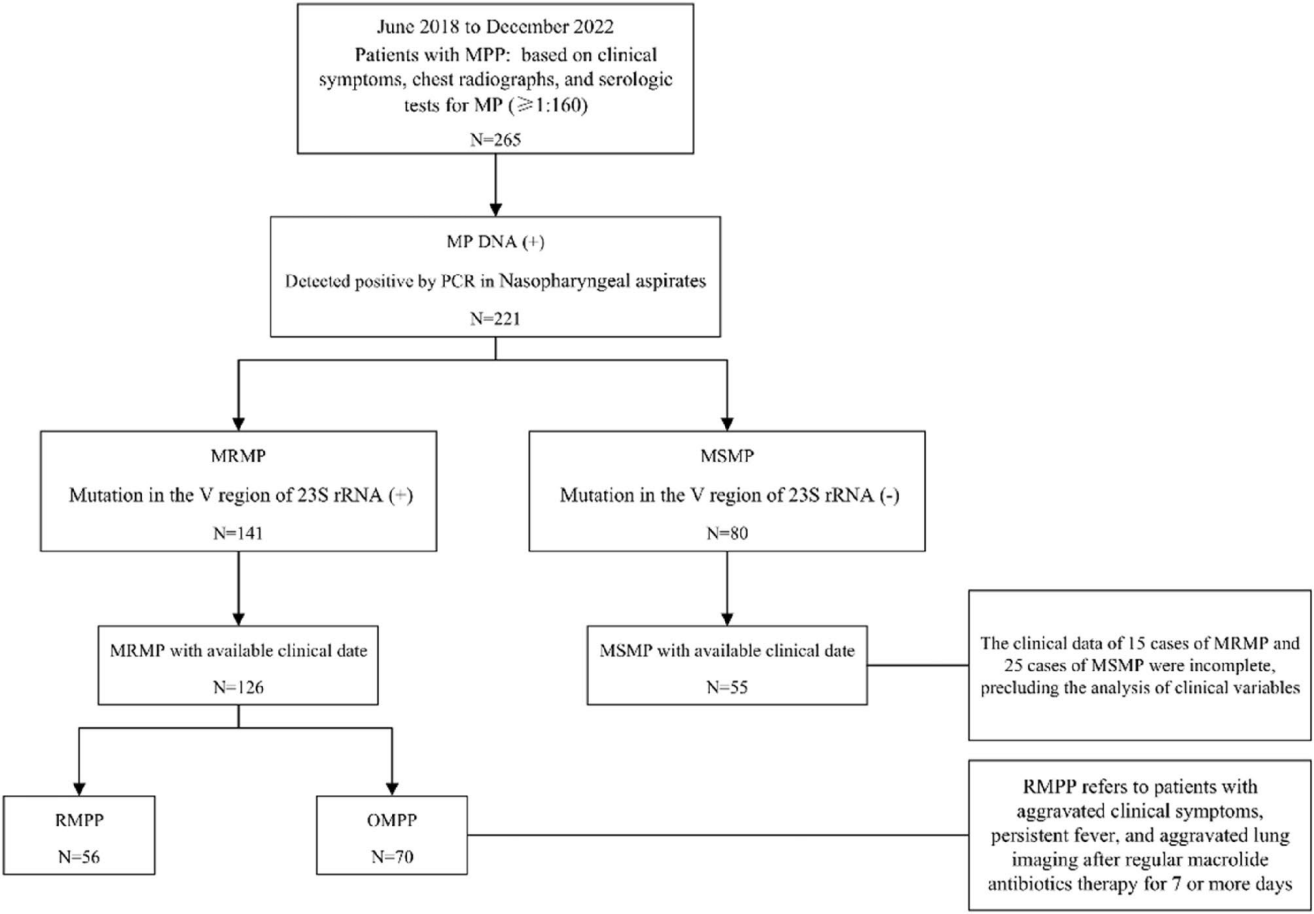
Flow chart of the study. MP: *Mycoplasma pneumoniae*; MRMP: Macrolide-Resistant *Mycoplasma pneumoniae*; MSMP: Macrolide-Sensitive *Mycoplasma pneumoniae*; RMPP: refractory *Mycoplasma pneumoniae* pneumonia; OMPP: ordinary *Mycoplasma pneumoniae* pneumonia.

A retrospective study was conducted among patients diagnosed with MPP at the Children's Hospital of Shanxi Province between June 2018 and December 2022. The diagnosis of MPP was based on clinical symptoms, signs of lower respiratory tract infection, chest radiographs, and serologic tests for MP (≥ 1:160). A total of 265 patients were included in the study. Based on the results of MP DNA detection and analysis of mutations of domain V of 23S rRNA using nasopharyngeal aspirates, the children with MPP were divided into two groups: MRMP (Mycoplasma-resistant MPP) and MSMP (Mycoplasma-sensitive MPP) groups (Fig. 1). To identify potential laboratory markers that could be used in routine clinical practice to distinguish RMPP from OMPP, patients in the MRMP group were further divided into RMPP and OMPP subgroups. RMPP referred patients who experienced worsened clinical symptoms, persistent fever, and aggravated lung imaging despite receiving regular macrolide antibiotic therapy for 7 or more days^21^. Notably, as of the data collection point, all patients had received standard macrolide treatment.

Patients with underlying conditions such as immunodeficiency, asthma, allergic diseases, or coinfection with viruses known to cause lower respiratory tract infections were excluded from the study.

### Specimens and clinical data

All samples, including nasopharyngeal aspirates and blood, were collected before treatment on the first day of admission. After admission, fiber bronchoscopic lavage was administered to 21 patients in the MRMP group and 12 patients in the MSMP group within 7 days of macrolide treatment. Additionally, 85 patients in the MRMP group and 26 in the MSMP group underwent fiber bronchoscopic lavage within 7–14 days post-macrolide treatment. Nasopharyngeal aspirates were subsequently collected. Nasopharyngeal aspirates were stored at − 80 °C until they were used for PCR assays. The demographic characteristics of patients who tested positive for MP DNA were collected. Additionally, electronic medical records, including physical examination findings, imaging results, clinical course, and laboratory data, were reviewed and analyzed. The study protocol conformed to the ethical guidelines of the 1975 Declaration of Helsinki and was approved by the ethical committee of the Children’s Hospital of Shanxi Province (Protocol number: 2021020). Written informed consent to participate in this study was provided by the participant's legal guardian.

### Detection methods

The Detection of MP DNA was performed by real-time PCR on nasopharyngeal aspirates using the MP DNA Fluorescence PCR Diagnostic Kit (Sansure, Hunan, China). PCR was carried out using Bio-Rad CFX-96 Real-Time PCR instruments, with a reaction volume of 50 μl comprising 10 μl of DNA extraction, 0.4 μl of internal standard, 2 μl of enzyme mix, and 38 μl of reaction solution. The PCR reactions were carried out under the following conditions: 50 °C for 2 min, 94 °C for 5 min, followed by 45 cycles of denaturation at 94 °C for 15 s and annealing at 57 °C for 30 s. The results were reported as copies/ml.

In addition to detecting MP DNA, the A2063G, and A2064G mutations were also identified using real-time PCR. For this purpose, the MP Drug-resistant Mutation Diagnostic Kit (Mole, Jiangsu, China) was employed. Similarly, the Bio-Rad CFX-96 Real-Time PCR instruments employed, utilizing a reaction volume of 20 μl consisting of 5 μl of DNA extraction, 0.5 μl of enzyme mix, and 14.5 μl of reaction solution. The PCR reactions were carried out under the following conditions: 50 °C for 2 min, 95 °C for 5 min, followed by 40 cycles of denaturation at 91 °C for 15 s and annealing at 64 °C for 1 min.

Following standard procedures, white blood cell counts were performed on a Sysmex automated blood analyzer. The platelet-to-lymphocyte ratio (PLR), which represents the ratio of absolute platelet count to lymphocyte count, was calculated. Serum CRP levels were measured using standard methods with the Cobas6000 analyzer (Roche, Mannheim, Germany). Furthermore, the levels of community-acquired respiratory distress syndrome (CARDS) toxin and interleukin (IL) 18 in nasopharyngeal aspirate were detected using enzyme-linked immunosorbent assay kits (Enzyme Linked, Shanghai, China) according to the manufacturer’s instructions.

### Statistical analyses

Continuous variables were described as medians and interquartile ranges, and categorical variables were presented as counts and percentages. As appropriate, data were analyzed statistically using chi-square analysis, Kruskal–Wallis H-test, and Mann–Whitney U-test. All data were analyzed using the SPSS software version 25.0 (IBM, Armonk, NY, USA), and *P* < 0.05 was considered statistically significant.

## Results

### General information and the proportion of MRMP

A total of 265 patients were included in the study, of whom 221 tested positive for MP DNA, accounting for 83.4% of the total sample. Among these positive cases, 63.8% (141/221) had the A2063G or A2064G mutation in the V region of 23S rRNA, while 36.2% (80/221) tested negative for these mutations. However, due to incomplete clinical data for 15 cases of mutation-positive and 25 cases of mutation-negative patients, it was impossible to analyze these individuals' clinical variables. In summary, a total of 181 cases with a positive MP DNA screening test were eventually included in the analysis, with 126 cases belonging to the MRMP group and 55 cases to the MSMP group (Fig. 1).

### Clinical characteristic and laboratory findings of MRMP and MSMP patients

There were no significant differences in gender (*P* = 0.933), age (*P* = 0.780), or length of stay (*P* = 0.389) between the MRMP and MSMP groups. The results of chest radiography (*P* = 0.698) and laboratory findings, including WBC (*P* = 0.212), Lymphocyte (*P* = 0.075), and Platelet count (*P* = 0.536) upon admission, were not statistically significant. However, the entire febrile days (median and interquartile ranges) were longer in the MRMP group compared to the MSMP group (8 [6–11] vs. 6 [4–10]; *P* = 0.004). Additionally, the CRP level (median and interquartile ranges) of patients with MRMP was significantly higher than those in the MSMP group (28.33 [18.95–40.33] vs. 21.00 [15.33–27.00]; *P* < 0.001). For details, see Table 1.

**Table 1:** Clinical characteristic and laboratory findings of MRMP and MSMP patients.

	MRMP (n = 126)	MSMP (n = 55)	*P*
Male/female	65/61	28/27	0.933
Age (years)	5.00 ± 3.26	5.15 ± 3.64	0.780
Age groups [n (%)]			0.909
< 1 year	9 (7.14%)	5 (3.97%)	
1 year and above	26 (20.63%)	13 (10.32%)	
3 years and above	49 (38.89%)	19 (15.08%)	
7–14 years	42 (33.33%)	18 (14.29%)	
Hospital stays (day)	9 (6–14%)	7 (5–14)	0.389
Total febrile days	8 (6–11)	6 (4–10)	0.004*
Chest x-ray			
Patchy high-density lesion/consolidation [n (%)]	45 (35.71%)	18 (32.73%)	0.698
Laboratory findings			
White blood cell (× 10^9^/L)	8.44 (6.54–12.71)	8.21 (6.30–10.04)	0.212
Lymphocyte (× 10^9^/L)	2.56 (2.18–3.28)	2.86 (2.18–4.18)	0.075
Platelet (× 10^9^/L)	272 (213–336)	258 (194–320)	0.536
PLR	96.03 (76.54–129.33)	87.46 (69.83–118.40)	0.047
C-reactive protein (mg/L)	28.33 (18.95–40.33)	21.00 (15.33–27.00)	< 0.001*

Data are given as mean ± SD, median (IQR) or n (%).

MRMP: Macrolide-Resistant *Mycoplasma pneumoniae*; MSMP*:* Macrolide-Sensitive *Mycoplasma pneumoniae*; PLR: platelet-to-lymphocyte ratio.

*Bold values indicate statistically significant *P* < 0.05.

### Comparison of MP DNA load in MRMP and MSMP patients

We analyzed the MP DNA load of all patients upon admission and found that the MRMP group had a higher mean [± SD] MP DNA load than the MSMP group (6.13 [± 1.23] vs. 5.64 [± 1.86]; *P* = 0.037). Within 7 days after treatment with macrolides, fiberbronchoscopic lavage was performed on 21 patients in the MRMP group, and 12 patients in the MSMP group, and their MP DNA loads were simultaneously measured. The results revealed significant differences between the two groups: (6.12 ± 1.27 vs. 4.55 ± 2.02, *P* = 0.010). Fibrobronchoscopic lavage was also conducted on 85 patients in the MRMP group and 26 patients in the MSMP group within 7 to 14 days after macrolide treatment, yielding the following MP DNA load measurements: (4.69 ± 1.40 vs. 3.55 ± 1.52, *P* < 0.001). Throughout the entire disease course (from admission to the first fiberbronchoscopy), the MRMP group consistently exhibited higher MP DNA loads compared to the MSMP group, and the decline in MP DNA load appeared to be slower in the MRMP group than in the MSMP group (Table 2).

**Table 2:** Comparison of MP DNA load in MRMP and MSMP patients with different courses of disease.

Course of disease	MRMP		MSMP		*P*
	Number of patients	log_10_ MP DNA	Number of patients	log_10_ MP DNA	
Upon Admission	126	6.13 ± 1.23	55	5.64 ± 1.86	0.037*
≤ 7 d	21	6.12 ± 1.27	12	4.55 ± 2.02	0.010*
> 7 d	85	4.69 ± 1.40	26	3.55 ± 1.52	< 0.001*

Data are given as mean ± SD.

MP: *Mycoplasma pneumoniae*; MRMP: macrolide-resistant *Mycoplasma pneumoniae*; MSMP*:* macrolide-sensitive *Mycoplasma pneumoniae.*

*Bold values indicate statistically significant *P* < 0.05.

### Comparison of CRP, CARDS toxin and IL-18 in nasopharyngeal aspirate in RMPP and OMPP patients

Based on clinical symptomatology, the MRMP group was subdivided into two subgroups: the RMPP group (n = 56) and the OMPP group (n = 70) (Fig. 1). The CRP, CARDS toxin and IL-18 levels (median and interquartile ranges) were measured in both groups and presented in Table 3. When comparing the OMPP subgroup to the RMPP subset, it was observed that the RMPP subgroup displayed significantly elevated levels of IL-18 (141.45 [91.93–182.32] vs. 59.84 [32.98–94.14]; *P* < 0.001) and CARDS toxin (42.73 [25.16–67.06] vs. 32.91 [16.22–47.43]; *P* = 0.006) in the nasopharyngeal aspirate and serum CRP (36.33 [27.50–53.83] vs. 22.83 [14.67–29.75]; *P* < 0.001). As shown in Table 1, there were no significant differences in gender and age between the two groups (*P* = 0.690; *P* = 0.721). Furthermore, in terms of laboratory examinations, there were also no significant differences between the two groups in white blood cell count, lymphocyte count, platelet count, and PLR (*P* = 0.192; *P* = 0.330;* P* = 0.562; *P* = 0.673).

**Table 3:** Comparison of CRP, CARDS toxin and IL-18 in nasopharyngeal aspirate in RMPP and OMPP patients.

	RMPP (n = 56)	OMPP (n = 70)	*P*
Boy/girl	30/26	35/35	0.690
Age (years)	5.11 ± 3.41	4.90 ± 3.16	0.721
White blood cell (× 10^9^/L)	9.89 (7.18–13.43)	8.31 (6.48–12.44)	0.192
Lymphocyte (× 10^9^/L)	2.58 (2.21–3.28)	2.56 (2.06–3.39)	0.330
Platelet (× 10^9^/L)	275 (219–351)	273 (212–325)	0.562
PLR	97.93 (81.50–134.69)	96.03 (74.00–126.17)	0.673
C-reactive protein (mg/L)	36.33 (27.50–53.83)	22.83 (14.67–29.75)	< 0.001*
IL-18 (pg/mL)	141.45 (91.93–182.32)	59.84 (32.98–94.14)	< 0.001*
CARDS toxin (pg/mL)	42.73 (25.16–67.06)	32.91 (16.22–47.43)	0.006*

Data are given as mean ± SD, median (IQR).

RMPP: refractory *Mycoplasma pneumoniae* pneumonia; OMPP: ordinary *Mycoplasma pneumoniae* pneumonia; PLR: platelet-to-lymphocyte ratio.

*Bold values indicate statistically significant p < 0.05.

## Discussion

MP is a significant pathogen in the context of community-acquired pneumonia among children. It is primarily transmitted through the respiratory tract and exhibits periodic pandemic outbreaks every 3 to 5 years^2^. Currently, macrolides are the recommended first-line treatment for MP infection. However, the widespread use of these drugs in patients with various types of infections has led to an increased incidence of macrolide resistance in recent years^3,22^. In today's globalized world, the widespread presence of drug-resistant organisms underscores the growing severity of macrolide resistance in MP. Currently, countries worldwide are monitoring the prevalence of macrolide resistance in MP. Europe and America generally show lower resistance rates, whereas East Asia faces the most severe and steadily increasing resistance trends^9–11,23–26^. Unlike other bacteria, MP poses unique challenges in routine clinical practice due to its slow growth characteristics and the lack of practical phenotypic drug susceptibility testing. Consequently, there is a dearth of routine cumulative antimicrobial susceptibility data to guide empirical treatment, leading clinicians to initiate antimicrobial therapy without knowledge of macrolide resistance rates^6,14,16,24,27^. Hence, it becomes crucial to comprehend the clinical features, antibacterial therapy, and outcomes in populations with drug-resistant strains.

Macrolide resistance in MP is primarily attributed to mutations in the 23S rRNA gene. In China, the A2063G mutation has emerged as the predominant cause of macrolide resistance in MP, with a detection rate exceeding 95%^17,18^. Therefore, in our study, we employed the detection of A2063G and A2064G mutations to identify MRMP. Several Chinese studies have reported a high prevalence of MRMP strains among patients with respiratory infections, ranging from 60 to 92%^10,28–31^. Consistent with previous studies findings, the proportion of macrolide resistance in pediatric patients with MPP was 63.8% in the current study. These results further corroborate the significant issue of macrolide drug resistance in China. In the current study, there were no significant differences in gender composition, age, and total length of stay between the group of MRMP and MSMP. The chest radiography results and laboratory findings, including WBC, lymphocyte, and platelet count upon admission, showed no statistical significance. However, the total number of febrile days was longer in the MRMP group compared to the MSMP group. Additionally, the level of CRP in patients with MRMP was significantly higher than in the MSMP group. When analyzing the MP DNA load of all patients throughout the entire disease course (from admission to the first fiberbronchoscopy after treatment with macrolides), the MRMP group consistently exhibited higher MP DNA loads compared to the MSMP group, and the decline in MP DNA load appeared to be slower in the MRMP group than in the MSMP group. A high MP DNA load may contribute to more severe airway damage, potentially associated with the prolonged duration of fever observed in the MRMP group^32^.

Some researchers have suggested that continuing the use of macrolide remains effective in most cases of MRMP patients^33^. In this study, the clinical characteristics and certain laboratory findings of both groups also affirmed the therapeutic effect of macrolide on MRMP patients. One possible explanation is the non-antibacterial action of macrolide. Numerous studies have shown that certain macrolides have broad immunomodulatory effects on mammalian cells, both in vivo and in vitro^34–37^. These effects limit neutrophil-mediated tissue damage, reduce mucus viscosity, inhibit angiogenesis, and support the fundamental principles of using macrolide to treat chronic inflammatory airway diseases^38^. In this study, although the MRMP group consistently exhibited higher MP DNA loads compared to the MSMP group during different courses of the disease, the immunomodulatory effects of macrolide may improve the clinical symptoms of these MRMP patients. Previous reports on the clinical course of MRMP have suggested that resistant strains do not increase the severity of the disease^39,40^. However, MRMP patients had more extended febrile periods and higher levels of CRP, indicating lower microbiological and clinical efficacy of macrolides in treating them. Therefore, the additive-sensitive anti-MP drugs for eligible patients may still be beneficial in improving treatment outcomes. Additionally, appropriate antibiotics may be necessary to eradicate MP and prevent community transmission, recurrence, and complications such as asthma.

Moreover, an alternative therapeutic approach involves the administration of systemic corticosteroids in addition to antimicrobials, particularly in pediatric patients with RMPP. In addition to respiratory symptoms, RMPP infection can also cause other systemic manifestations, suggesting that the excessive host immune response, including innate immunity and adaptive immunity, plays a vital role in the pathogenesis of RMPP infection^41^. Therefore, the next challenge lies in the timely diagnosis and proactive management of RMPP. Community-acquired respiratory distress syndrome (CARDS) toxin is a vital toxin produced by MP. Previous research has demonstrated that primates exposed to MP and CARDS toxin exhibited similar histopathological changes in the lung tissues^42^. The CARDS toxin can induce NLRP3 (NLR-family, leucine-rich repeat protein 3) inflammasome activation, resulting in the secretion of interleukin-1β (IL-1β) and interleukin-18 (IL-18) by macrophages. This process can potentially impair the integrity of the respiratory barrier and contribute to cellular damage^43–45^. We further divided the MRMP group into the RMPP and the OMPP groups based on clinical symptoms. Our data revealed that the RMPP subgroup displayed significantly elevated levels of IL-18 and CARD toxins in the nasopharyngeal aspirate. This observation suggests that CARDS TX may be crucial in inducing a pronounced inflammatory response in children diagnosed with RMPP. However, it is worth noting that in China, most hospital laboratories do not routinely measure IL-18 and CARDS toxin levels in nasopharyngeal aspirates. Therefore, we proceeded to analyze several clinically established laboratory parameters. Several studies have found elevated serum CRP levels in RMPP patients^2,46^. Consistent with previous research, we observed that the RMPP subgroup displayed significantly higher levels of serum CRP. This finding suggests an excessive inflammatory reaction in RMPP patients, and serum CRP is a reliable indicator for predicting RMPP progression.

Furthermore, platelet-to-lymphocyte ratio (PLR), the most commonly used and simplest biomarker reflecting the immune system, refers to the ratio of platelets to lymphocytes in peripheral blood and can reflect the body's systemic inflammatory state. A previous study demonstrated a significant increase in PLR during acute exacerbation of chronic obstructive pulmonary disease, indicating its potential to assess the severity of inflammatory diseases^47^. However, our study found no significant difference in PLR between RMPP and OPMM patients, suggesting that PLR, as a nonspecific biomarker, may not be a reliable indicator for predicting inflammatory response in pediatric patients. In summary, nasopharyngeal aspirate IL-18, CARD toxins, and serum CRP may be promising biomarkers for predicting RMPP. However, for the clinical application of nasopharyngeal aspirate IL-18 and CARD toxins, standardized detection procedures are required to be established.

Limited by the retrospective design of this study, most data on MP infection prevalence were collected simultaneously, making it unsuitable for observing the dynamic nature of the infection. Furthermore, the search for mutations in ribosomal proteins L4 was not conducted in this study because the resistance to macrolides in China has primarily been attributed to 23S rRNA mutation, potentially leading to an underestimation of the proportion of MRMP. Additionally, being a single-center study, our findings only partially reflect MP infections in a provincial city, and thus generalizing our results to the broader pediatric population in China may introduce selection bias. Thus, multi-center studies with increased sample sizes would be instrumental in further validating and consolidating our findings.

In summary, this study reveals that patients with MRMP exhibit relatively severe signs and symptoms. The MP DNA load is higher, and the decline after macrolide antibiotic treatment is slow, indicating reduced drug efficacy. Tracking the duration of fever, MP DNA load, and the degree of decrease after macrolide drug treatment in children infected with MP is crucial for identifying MRMP. Moreover, early prediction of RMPP is essential for appropriate patient management. The study results suggest that nasopharyngeal aspirate interleukin-18 (IL-18), CARD toxins, and serum CRP may serve as valuable markers for predicting RMPP. In conclusion, early recognition of RMPP is imperative for prognosis improvement, and the identification of MRMP along with proinflammatory cytokines (IL-18, CARDS toxins, and CRP) stands out as potential markers.

## Data Availability

All data generated or analyzed during this study are included in this published article. Further inquiries can be directed to the corresponding author.
